# 2-(2-Fluoro­phen­yl)-3-methyl­sulfanyl-5-phenyl-1-benzo­furan

**DOI:** 10.1107/S1600536813009768

**Published:** 2013-04-13

**Authors:** Hong Dae Choi, Pil Ja Seo, Uk Lee

**Affiliations:** a Department of Chemistry, Dongeui University, San 24 Kaya-dong, Busanjin-gu, Busan 614-714, Republic of Korea; b Department of Chemistry, Pukyong National University, 599-1 Daeyeon 3-dong, Nam-gu, Busan 608-737, Republic of Korea

## Abstract

In the title compound, C_21_H_15_FOS, the dihedral angles between the mean plane [r.m.s. deviation = 0.041 (1) Å] of the benzo­furan fragment and the pendant 2-fluoro­phenyl and phenyl rings are 46.09 (3) and 24.34 (5)°, respectively. In the crystal, mol­ecules are linked by weak C—H⋯π inter­actions, forming a three-dimensional network.

## Related literature

For background information and the crystal structures of related compounds, see: Choi *et al.* (2006 ▶); Seo *et al.* (2011 ▶).
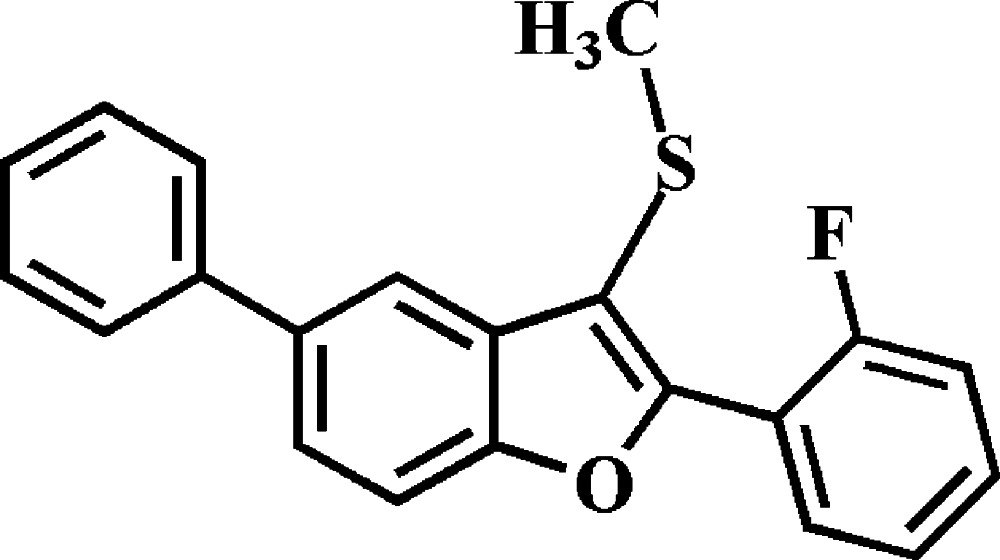


## Experimental

### 

#### Crystal data

C_21_H_15_FOS
*M* *_r_* = 334.39
Monoclinic, 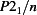

*a* = 11.1257 (2) Å
*b* = 7.4232 (1) Å
*c* = 19.4212 (3) Å
β = 97.319 (1)°
*V* = 1590.90 (4) Å^3^
*Z* = 4
Mo *K*α radiation
μ = 0.22 mm^−1^
*T* = 173 K
0.27 × 0.19 × 0.14 mm

#### Data collection

Bruker APEXII CCD diffractometer
Absorption correction: multi-scan (*SADABS*; Bruker, 2009 ▶) *T*_min_ = 0.689, *T*_max_ = 0.746
28795 measured reflections
3967 independent reflections
3190 reflections with *I* > 2σ(*I*)
*R*_int_ = 0.040

#### Refinement

*R*[*F* ^2^ > 2σ(*F* ^2^)] = 0.037
*wR*(*F* ^2^) = 0.101
*S* = 1.05
3967 reflections
218 parameters
H-atom parameters constrained
Δρ_max_ = 0.26 e Å^−3^
Δρ_min_ = −0.26 e Å^−3^

### 

Data collection: *APEX2* (Bruker, 2009 ▶); cell refinement: *SAINT* (Bruker, 2009 ▶); data reduction: *SAINT*; program(s) used to solve structure: *SHELXS97* (Sheldrick, 2008 ▶); program(s) used to refine structure: *SHELXL97* (Sheldrick, 2008 ▶); molecular graphics: *ORTEP-3* for Windows (Farrugia, 2012 ▶) and *DIAMOND* (Brandenburg, 1998 ▶); software used to prepare material for publication: *SHELXL97*.

## Supplementary Material

Crystal structure: contains datablock(s) global, I. DOI: 10.1107/S1600536813009768/ds2230sup1.cif

Structure factors: contains datablock(s) I. DOI: 10.1107/S1600536813009768/ds2230Isup2.hkl

Supplementary material file. DOI: 10.1107/S1600536813009768/ds2230Isup3.cml

Additional supplementary materials:  crystallographic information; 3D view; checkCIF report

## Figures and Tables

**Table 1:** Hydrogen-bond geometry (Å, °) *Cg*1 and *Cg*2 are the C9–C14 phenyl and 2-fluoro­phenyl rings, respectively.

*D*—H⋯*A*	*D*—H	H⋯*A*	*D*⋯*A*	*D*—H⋯*A*
C10—H10⋯*Cg*1^i^	0.95	2.82	3.682 (2)	131
C14—H14⋯*Cg*2^ii^	0.95	2.71	3.528 (2)	145

Symmetry codes: (i) 
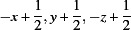
; (ii) 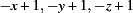.

**Fig. 1.**
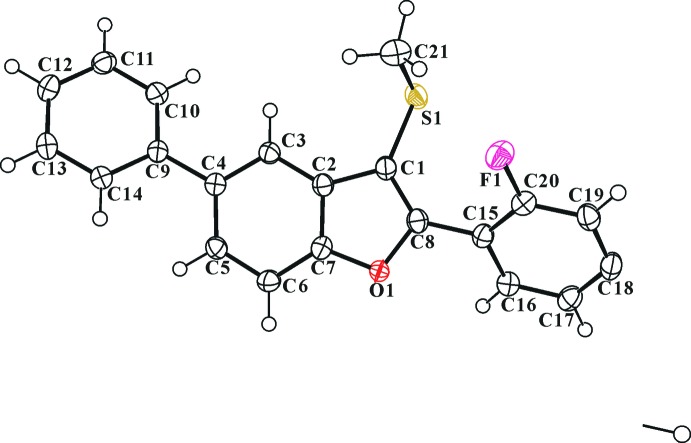
The molecular structure of the title compound with the atom numbering scheme. Displacement ellipsoids are drawn at the 50% probability level. H atoms are presented as small spheres of arbitrary radius.

**Fig. 2.**
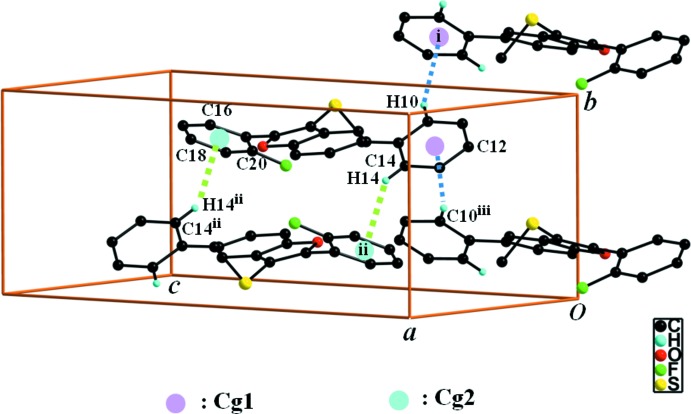
A view of the C–H..π interactions (dotted lines) in the crystal structure of the title compound. H atoms non-participating in hydrogen-bonding were omitted for clarity. [Symmetry codes: (i) - x + 1/2, y + 1/2, - z + 1/2; (ii) - x + 1, - y + 1, - z + 1; (iii) - x + 1/2, y - 1/2, - z + 1/2.]

## supplementary crystallographic information

### Comment

As a part of our continuing study of 3-methylsulfanyl-5-phenyl-1-benzofuran derivatives containing phenyl (Choi *et al.*, 2006) and 3-fluorophenyl (Seo *et al.*, 2011) substituents in 2-position, we report herein the crystal structure of the title compound.

In the title molecule (Fig. 1), the benzofuran unit is essentially planar, with a mean deviation of 0.041 (1) Å from the least-squares plane defined by the nine constituent atoms. The dihedral angles between the mean plane of the benzofuran fragment and the pendant 2-fluorophenyl and phenyl rings are 46.09 (3) and 24.34 (5), respectively. In the crystal structure (Fig. 2), molecules are connected by weak intermolecular C–H···π interactions (Table 1, Cg1 and Cg2 are the centroids of the C9–C14 phenyl ring and the C15-C20 2-fluorophenyl ring, respectively).

### Experimental

Zinc chloride (218 mg, 1.6 mmol) was added to a stirred solution of 4-phenylphenol (272 mg, 1.6 mmol) and 2-chloro-2-methylsulfanyl-2'-fluoroacetophenone (350 mg, 1.6 mmol) in dichloromethene (20 mL) at room temperature, and stirring was continued at the same temperature for 40 min. The reaction was quenched by the addition of water and the organic layer was separated, dried over magnesium sulfate, filtered and concentrated at reduced pressure. The residue was purified by column chromatography (hexane-benzene, 5:2 v/v) to afford the title compound as a colorless solid [yield 51%, m.p. 368-369 K; *R*_f_ = 0.63 (hexane-benzene, 5:2 v/v)]. Single crystals suitable for X-ray diffraction were prepared by slow evaporation of a solution of the title compound in acetone at room temperature.

### Refinement

All H atoms were positioned geometrically and refined using a riding model, with C–H = 0.95 Å for aryl and 0.98 Å for methyl H atoms. *U*_iso_(H) = 1.2*U*_eq_(C) for aryl and 1.5*U*_eq_(C) for methyl H atoms. The positions of methyl hydrogens were optimized rotationally.

### Crystal data

**Table:**

C_21_H_15_FOS	*F*(000) = 696
*M**_r_* = 334.39	*D*_x_ = 1.396 Mg m^−^^3^
Monoclinic, *P*2_1_/*n*	Melting point = 368–369 K
Hall symbol: -P 2yn	Mo *K*α radiation, λ = 0.71073 Å
*a* = 11.1257 (2) Å	Cell parameters from 7139 reflections
*b* = 7.4232 (1) Å	θ = 2.2–27.4°
*c* = 19.4212 (3) Å	µ = 0.22 mm^−^^1^
β = 97.319 (1)°	*T* = 173 K
*V* = 1590.90 (4) Å^3^	Block, colourless
*Z* = 4	0.27 × 0.19 × 0.14 mm

### Data collection

**Table:**

Bruker APEXII CCD diffractometer	3967 independent reflections
Radiation source: rotating anode	3190 reflections with *I* > 2σ(*I*)
Graphite multilayer monochromator	*R*_int_ = 0.040
Detector resolution: 10.0 pixels mm^-1^	θ_max_ = 28.4°, θ_min_ = 2.0°
φ and ω scans	*h* = −14→14
Absorption correction: multi-scan (*SADABS*; Bruker, 2009)	*k* = −9→9
*T*_min_ = 0.689, *T*_max_ = 0.746	*l* = −25→25
28795 measured reflections	

### Refinement

**Table:**

Refinement on *F*^2^	Primary atom site location: structure-invariant direct methods
Least-squares matrix: full	Secondary atom site location: difference Fourier map
*R*[*F*^2^ > 2σ(*F*^2^)] = 0.037	Hydrogen site location: difference Fourier map
*wR*(*F*^2^) = 0.101	H-atom parameters constrained
*S* = 1.05	*w* = 1/[σ^2^(*F*_o_^2^) + (0.0483*P*)^2^ + 0.4925*P*] where *P* = (*F*_o_^2^ + 2*F*_c_^2^)/3
3967 reflections	(Δ/σ)_max_ = 0.001
218 parameters	Δρ_max_ = 0.26 e Å^−^^3^
0 restraints	Δρ_min_ = −0.26 e Å^−^^3^

### Special details

**Table:**

Geometry. All esds (except the esd in the dihedral angle between two l.s. planes) are estimated using the full covariance matrix. The cell esds are taken into account individually in the estimation of esds in distances, angles and torsion angles; correlations between esds in cell parameters are only used when they are defined by crystal symmetry. An approximate (isotropic) treatment of cell esds is used for estimating esds involving l.s. planes.
Refinement. Refinement of F^2^ against ALL reflections. The weighted R-factor wR and goodness of fit S are based on F^2^, conventional R-factors R are based on F, with F set to zero for negative F^2^. The threshold expression of F^2^ > 2sigma(F^2^) is used only for calculating R-factors(gt) etc. and is not relevant to the choice of reflections for refinement. R-factors based on F^2^ are statistically about twice as large as those based on F, and R- factors based on ALL data will be even larger.

### Fractional atomic coordinates and isotropic or equivalent isotropic displacement parameters (Å^2^)

**Table:**

	*x*	*y*	*z*	*U*_iso_*/*U*_eq_	
S1	0.16737 (3)	0.90172 (5)	0.526490 (19)	0.03354 (12)	
F1	0.19589 (8)	0.59691 (12)	0.63666 (4)	0.0364 (2)	
O1	0.50485 (9)	0.72718 (13)	0.56484 (5)	0.0267 (2)	
C1	0.31237 (13)	0.81153 (18)	0.52659 (7)	0.0251 (3)	
C2	0.37725 (12)	0.79253 (18)	0.46729 (7)	0.0233 (3)	
C3	0.34570 (12)	0.80710 (18)	0.39580 (7)	0.0242 (3)	
H3	0.2662	0.8429	0.3772	0.029*	
C4	0.43212 (12)	0.76849 (17)	0.35187 (7)	0.0221 (3)	
C5	0.55052 (12)	0.72200 (19)	0.38149 (7)	0.0258 (3)	
H5	0.6099	0.6998	0.3515	0.031*	
C6	0.58376 (13)	0.7074 (2)	0.45239 (7)	0.0282 (3)	
H6	0.6638	0.6757	0.4716	0.034*	
C7	0.49417 (13)	0.74160 (18)	0.49375 (7)	0.0244 (3)	
C8	0.39147 (13)	0.76675 (18)	0.58301 (7)	0.0244 (3)	
C9	0.39863 (12)	0.76923 (17)	0.27537 (7)	0.0218 (3)	
C10	0.29923 (12)	0.86819 (19)	0.24410 (7)	0.0266 (3)	
H10	0.2541	0.9401	0.2721	0.032*	
C11	0.26579 (13)	0.8628 (2)	0.17297 (7)	0.0298 (3)	
H11	0.1976	0.9300	0.1527	0.036*	
C12	0.33101 (14)	0.7602 (2)	0.13126 (7)	0.0327 (3)	
H12	0.3076	0.7561	0.0825	0.039*	
C13	0.43046 (14)	0.6637 (2)	0.16105 (7)	0.0325 (3)	
H13	0.4764	0.5946	0.1326	0.039*	
C14	0.46343 (13)	0.66746 (19)	0.23219 (7)	0.0265 (3)	
H14	0.5315	0.5994	0.2520	0.032*	
C15	0.38210 (13)	0.75223 (17)	0.65736 (7)	0.0241 (3)	
C16	0.47502 (13)	0.81460 (18)	0.70674 (7)	0.0263 (3)	
H16	0.5452	0.8668	0.6918	0.032*	
C17	0.46672 (14)	0.80178 (19)	0.77690 (7)	0.0299 (3)	
H17	0.5302	0.8471	0.8097	0.036*	
C18	0.36603 (14)	0.7229 (2)	0.79949 (7)	0.0303 (3)	
H18	0.3602	0.7153	0.8478	0.036*	
C19	0.27373 (13)	0.65489 (19)	0.75195 (7)	0.0284 (3)	
H19	0.2050	0.5985	0.7671	0.034*	
C20	0.28393 (13)	0.67099 (18)	0.68235 (7)	0.0262 (3)	
C21	0.07375 (16)	0.7190 (3)	0.48970 (9)	0.0480 (4)	
H21A	0.1000	0.6826	0.4455	0.072*	
H21B	−0.0110	0.7585	0.4818	0.072*	
H21C	0.0812	0.6167	0.5218	0.072*	

### Atomic displacement parameters (Å^2^)

**Table:**

	*U*^11^	*U*^22^	*U*^33^	*U*^12^	*U*^13^	*U*^23^
S1	0.0334 (2)	0.0392 (2)	0.0296 (2)	0.01220 (16)	0.01019 (15)	0.00356 (15)
F1	0.0344 (5)	0.0430 (5)	0.0304 (5)	−0.0135 (4)	−0.0006 (4)	0.0033 (4)
O1	0.0262 (5)	0.0344 (5)	0.0195 (5)	−0.0012 (4)	0.0030 (4)	0.0012 (4)
C1	0.0285 (7)	0.0251 (7)	0.0223 (6)	0.0019 (5)	0.0058 (5)	0.0005 (5)
C2	0.0254 (7)	0.0215 (6)	0.0234 (6)	0.0003 (5)	0.0041 (5)	−0.0007 (5)
C3	0.0247 (7)	0.0249 (6)	0.0231 (6)	0.0027 (5)	0.0036 (5)	0.0005 (5)
C4	0.0249 (7)	0.0194 (6)	0.0222 (6)	−0.0018 (5)	0.0038 (5)	−0.0001 (5)
C5	0.0234 (7)	0.0299 (7)	0.0250 (7)	−0.0019 (5)	0.0059 (5)	−0.0004 (5)
C6	0.0222 (7)	0.0361 (8)	0.0259 (7)	−0.0013 (6)	0.0017 (5)	0.0016 (6)
C7	0.0271 (7)	0.0249 (7)	0.0210 (6)	−0.0033 (5)	0.0026 (5)	−0.0003 (5)
C8	0.0272 (7)	0.0227 (6)	0.0241 (7)	−0.0016 (5)	0.0060 (5)	−0.0004 (5)
C9	0.0232 (6)	0.0210 (6)	0.0216 (6)	−0.0036 (5)	0.0040 (5)	−0.0008 (5)
C10	0.0264 (7)	0.0268 (7)	0.0267 (7)	0.0008 (5)	0.0040 (5)	−0.0009 (5)
C11	0.0290 (7)	0.0319 (7)	0.0274 (7)	0.0024 (6)	−0.0008 (6)	0.0020 (6)
C12	0.0381 (8)	0.0391 (8)	0.0203 (7)	0.0000 (7)	0.0017 (6)	0.0001 (6)
C13	0.0350 (8)	0.0389 (8)	0.0248 (7)	0.0032 (6)	0.0083 (6)	−0.0048 (6)
C14	0.0255 (7)	0.0301 (7)	0.0242 (7)	0.0021 (6)	0.0043 (5)	−0.0014 (5)
C15	0.0294 (7)	0.0217 (6)	0.0214 (6)	0.0016 (5)	0.0037 (5)	0.0004 (5)
C16	0.0289 (7)	0.0245 (7)	0.0258 (7)	−0.0012 (5)	0.0042 (5)	−0.0001 (5)
C17	0.0349 (8)	0.0290 (7)	0.0247 (7)	0.0006 (6)	−0.0008 (6)	−0.0027 (5)
C18	0.0409 (8)	0.0300 (7)	0.0208 (7)	0.0061 (6)	0.0072 (6)	0.0014 (5)
C19	0.0321 (8)	0.0268 (7)	0.0279 (7)	0.0030 (6)	0.0105 (6)	0.0038 (5)
C20	0.0284 (7)	0.0237 (6)	0.0258 (7)	−0.0002 (5)	0.0011 (5)	0.0000 (5)
C21	0.0322 (9)	0.0693 (13)	0.0410 (10)	0.0020 (8)	−0.0013 (7)	−0.0052 (9)

### Geometric parameters (Å, º)

**Table:**

S1—C1	1.7464 (14)	C10—H10	0.9500
S1—C21	1.8013 (19)	C11—C12	1.383 (2)
F1—C20	1.3521 (16)	C11—H11	0.9500
O1—C7	1.3748 (16)	C12—C13	1.382 (2)
O1—C8	1.3840 (16)	C12—H12	0.9500
C1—C8	1.3565 (19)	C13—C14	1.3839 (19)
C1—C2	1.4420 (18)	C13—H13	0.9500
C2—C7	1.3887 (19)	C14—H14	0.9500
C2—C3	1.3922 (18)	C15—C20	1.3882 (19)
C3—C4	1.3941 (18)	C15—C16	1.3964 (19)
C3—H3	0.9500	C16—C17	1.381 (2)
C4—C5	1.4114 (19)	C16—H16	0.9500
C4—C9	1.4851 (18)	C17—C18	1.384 (2)
C5—C6	1.3837 (19)	C17—H17	0.9500
C5—H5	0.9500	C18—C19	1.386 (2)
C6—C7	1.381 (2)	C18—H18	0.9500
C6—H6	0.9500	C19—C20	1.3760 (19)
C8—C15	1.4649 (18)	C19—H19	0.9500
C9—C14	1.3956 (18)	C21—H21A	0.9800
C9—C10	1.4002 (19)	C21—H21B	0.9800
C10—C11	1.3847 (19)	C21—H21C	0.9800
			
C1—S1—C21	101.49 (8)	C10—C11—H11	119.8
C7—O1—C8	105.94 (10)	C13—C12—C11	119.49 (13)
C8—C1—C2	106.37 (12)	C13—C12—H12	120.3
C8—C1—S1	126.80 (11)	C11—C12—H12	120.3
C2—C1—S1	126.37 (10)	C12—C13—C14	120.26 (13)
C7—C2—C3	119.70 (13)	C12—C13—H13	119.9
C7—C2—C1	105.77 (12)	C14—C13—H13	119.9
C3—C2—C1	134.47 (13)	C13—C14—C9	121.28 (13)
C2—C3—C4	119.23 (13)	C13—C14—H14	119.4
C2—C3—H3	120.4	C9—C14—H14	119.4
C4—C3—H3	120.4	C20—C15—C16	116.78 (12)
C3—C4—C5	118.78 (12)	C20—C15—C8	122.31 (12)
C3—C4—C9	120.45 (12)	C16—C15—C8	120.87 (13)
C5—C4—C9	120.72 (12)	C17—C16—C15	121.21 (13)
C6—C5—C4	122.81 (13)	C17—C16—H16	119.4
C6—C5—H5	118.6	C15—C16—H16	119.4
C4—C5—H5	118.6	C16—C17—C18	120.04 (14)
C7—C6—C5	116.31 (13)	C16—C17—H17	120.0
C7—C6—H6	121.8	C18—C17—H17	120.0
C5—C6—H6	121.8	C17—C18—C19	120.26 (13)
O1—C7—C6	126.40 (13)	C17—C18—H18	119.9
O1—C7—C2	110.48 (12)	C19—C18—H18	119.9
C6—C7—C2	123.11 (13)	C20—C19—C18	118.43 (13)
C1—C8—O1	111.39 (11)	C20—C19—H19	120.8
C1—C8—C15	133.94 (13)	C18—C19—H19	120.8
O1—C8—C15	114.67 (12)	F1—C20—C19	117.78 (12)
C14—C9—C10	117.60 (12)	F1—C20—C15	118.92 (12)
C14—C9—C4	121.08 (12)	C19—C20—C15	123.24 (13)
C10—C9—C4	121.30 (12)	S1—C21—H21A	109.5
C11—C10—C9	120.98 (13)	S1—C21—H21B	109.5
C11—C10—H10	119.5	H21A—C21—H21B	109.5
C9—C10—H10	119.5	S1—C21—H21C	109.5
C12—C11—C10	120.38 (13)	H21A—C21—H21C	109.5
C12—C11—H11	119.8	H21B—C21—H21C	109.5
			
C21—S1—C1—C8	−112.43 (14)	C3—C4—C9—C14	−154.56 (13)
C21—S1—C1—C2	76.40 (14)	C5—C4—C9—C14	22.97 (19)
C8—C1—C2—C7	−1.73 (15)	C3—C4—C9—C10	23.80 (19)
S1—C1—C2—C7	170.91 (11)	C5—C4—C9—C10	−158.67 (13)
C8—C1—C2—C3	175.29 (15)	C14—C9—C10—C11	0.9 (2)
S1—C1—C2—C3	−12.1 (2)	C4—C9—C10—C11	−177.53 (13)
C7—C2—C3—C4	0.60 (19)	C9—C10—C11—C12	−0.6 (2)
C1—C2—C3—C4	−176.10 (14)	C10—C11—C12—C13	−0.4 (2)
C2—C3—C4—C5	−2.27 (19)	C11—C12—C13—C14	1.0 (2)
C2—C3—C4—C9	175.31 (12)	C12—C13—C14—C9	−0.7 (2)
C3—C4—C5—C6	2.1 (2)	C10—C9—C14—C13	−0.3 (2)
C9—C4—C5—C6	−175.46 (13)	C4—C9—C14—C13	178.15 (13)
C4—C5—C6—C7	−0.2 (2)	C1—C8—C15—C20	43.6 (2)
C8—O1—C7—C6	−177.46 (13)	O1—C8—C15—C20	−136.50 (13)
C8—O1—C7—C2	1.10 (14)	C1—C8—C15—C16	−138.77 (17)
C5—C6—C7—O1	176.77 (13)	O1—C8—C15—C16	41.10 (17)
C5—C6—C7—C2	−1.6 (2)	C20—C15—C16—C17	−2.4 (2)
C3—C2—C7—O1	−177.18 (11)	C8—C15—C16—C17	179.85 (13)
C1—C2—C7—O1	0.37 (15)	C15—C16—C17—C18	1.3 (2)
C3—C2—C7—C6	1.4 (2)	C16—C17—C18—C19	0.6 (2)
C1—C2—C7—C6	178.99 (13)	C17—C18—C19—C20	−1.2 (2)
C2—C1—C8—O1	2.52 (15)	C18—C19—C20—F1	177.08 (12)
S1—C1—C8—O1	−170.08 (10)	C18—C19—C20—C15	−0.1 (2)
C2—C1—C8—C15	−177.61 (14)	C16—C15—C20—F1	−175.28 (12)
S1—C1—C8—C15	9.8 (2)	C8—C15—C20—F1	2.4 (2)
C7—O1—C8—C1	−2.29 (15)	C16—C15—C20—C19	1.9 (2)
C7—O1—C8—C15	177.81 (11)	C8—C15—C20—C19	179.55 (13)

### Hydrogen-bond geometry (Å, º)

Cg1 and Cg2 are the C9–C14 phenyl and 2-fluorophenyl rings, respectively.

**Table:**

*D*—H···*A*	*D*—H	H···*A*	*D*···*A*	*D*—H···*A*
C10—H10···*Cg*1^i^	0.95	2.82	3.682 (2)	131
C14—H14···*Cg*2^ii^	0.95	2.71	3.528 (2)	145

Symmetry codes: (i) −*x*+1/2, *y*+1/2, −*z*+1/2; (ii) −*x*+1, −*y*+1, −*z*+1.
